# Associations of *ATR* and *CHEK1* Single Nucleotide Polymorphisms with Breast Cancer

**DOI:** 10.1371/journal.pone.0068578

**Published:** 2013-07-03

**Authors:** Wei-Yu Lin, Ian W. Brock, Dan Connley, Helen Cramp, Rachel Tucker, Jon Slate, Malcolm W. R. Reed, Sabapathy P. Balasubramanian, Lisa A. Cannon-Albright, Nicola J. Camp, Angela Cox

**Affiliations:** 1 Institute for Cancer Studies, Department of Oncology, CRUK/YCR Cancer Research Centre, University of Sheffield, Sheffield, United Kingdom; 2 Department of Animal and Plant Sciences, University of Sheffield, Sheffield, United Kingdom; 3 Academic Unit of Surgical Oncology, Department of Oncology, CRUK/YCR Cancer Research Centre, University of Sheffield, Sheffield, United Kingdom; 4 Department of Internal Medicine, University of Utah School of Medicine, Salt Lake City, Utah, United States of America; IFOM, Fondazione Istituto FIRC di Oncologia Molecolare, Italy

## Abstract

DNA damage and replication checkpoints mediated by the *ATR*-*CHEK1* pathway are key to the maintenance of genome stability, and both *ATR* and *CHEK1* have been proposed as potential breast cancer susceptibility genes. Many novel variants recently identified by the large resequencing projects have not yet been thoroughly tested in genome-wide association studies for breast cancer susceptibility. We therefore used a tagging SNP (tagSNP) approach based on recent SNP data available from the 1000 genomes projects, to investigate the roles of *ATR* and *CHEK1* in breast cancer risk and survival. *ATR* and *CHEK1* tagSNPs were genotyped in the Sheffield Breast Cancer Study (SBCS; 1011 cases and 1024 controls) using Illumina GoldenGate assays. Untyped SNPs were imputed using IMPUTE2, and associations between genotype and breast cancer risk and survival were evaluated using logistic and Cox proportional hazard regression models respectively on a per allele basis. Significant associations were further examined in a meta-analysis of published data or confirmed in the Utah Breast Cancer Study (UBCS). The most significant associations for breast cancer risk in SBCS came from rs6805118 in *ATR* (p=7.6x10^-5^) and rs2155388 in *CHEK1* (p=3.1x10^-6^), but neither remained significant after meta-analysis with other studies. However, meta-analysis of published data revealed a weak association between the *ATR* SNP rs1802904 (minor allele frequency is 12%) and breast cancer risk, with a summary odds ratio (confidence interval) of 0.90 (0.83-0.98) [p=0.0185] for the minor allele. Further replication of this SNP in larger studies is warranted since it is located in the target region of 2 microRNAs. No evidence of any survival effects of *ATR* or *CHEK1* SNPs were identified. We conclude that common alleles of *ATR* and *CHEK1* are not implicated in breast cancer risk or survival, but we cannot exclude effects of rare alleles and of common alleles with very small effect sizes.

## Introduction

Despite the successes of the genome-wide association studies (GWAS) in identifying breast cancer susceptibility loci, the level of breast cancer risk explained by these susceptibility loci remains modest [1]. Good coverage of genetic markers, usually single nucleotide polymorphisms (SNPs) that sufficiently represent the surrounding variants through linkage disequilibrium (LD), is essential to a successful screening of susceptibility loci. So far, not all regions have been well captured, and more genetic markers may be needed. For example, novel disease susceptibility loci have been identified from the meta-analysis of several GWAS together [2,3], suggesting that some loci are not well captured in the initial GWAS discovery stage, partly due to a lack of power. The 1000 genomes project is a good resource to complement the incomplete coverage of GWAS chips, in which low coverage whole-genome shotgun sequencing and targeted sequencing of known exons has been carried out on a large number of samples [4]. Many of the resulting novel variants have not yet been thoroughly tested in GWAS.

The *ATR*-*CHEK1* DNA damage response pathway is key for maintenance of genome stability. *ATR* recognises DNA single-strand breaks, and activates *CHEK1* to initiate cell cycle arrest and DNA replication inhibition, the repression of cyclin proteins, and the activation of Fanconi Anemia proteins for DNA repair [5]. The close cross-talk between *CHEK1* and *BRCA1*, the breast cancer tumour suppressor gene, at the G2/M checkpoint [6] suggests the potential importance of this pathway in breast cancer.

To date, a small number of gene-based association studies have been carried out to investigate the roles of *ATR* and *CHEK1* as breast cancer susceptibility genes, using a tagging SNP (tagSNPs) approach. In these studies a subset of representative SNPs were chosen from previous reference panels that are less complete than those currently available [7–12]. Inconclusive evidence was reported for the *ATR* and *CHEK1* SNPs in association with breast cancer risk [7–12]. In addition, associations with survival following breast cancer diagnosis are examined in two of these studies, and null findings were reported [8,12]. Here, we have carried out a more detailed study of the role of the *ATR* and *CHEK1* in breast cancer risk and survival based on the more complete resources now available from the 1000 genomes project.

## Materials and Methods

### Ethics Statement

All Sheffield and Utah participants gave written informed consent for the collection of data and blood samples. The Sheffield and Utah studies were approved by South Sheffield Research Ethics Committee and University of Utah Institutional Review Board, respectively.

### Study populations

The Sheffield Breast Cancer Study (SBCS) recruited histologically confirmed breast cancer patients from surgical outpatient clinics in the Royal Hallamshire Hospital, Sheffield and Rotherham District Hospital from 1998 to 2005. Women aged 50-60 years attending the mammography breast screening service in Sheffield between October 2000 and January 2004 were drawn as control subjects if there was no evidence of breast lesions in their mammograms. Samples from 1011 cases and 1024 controls were available for genotyping. Cases and controls were all resident in the Sheffield area and of northern European ancestry. Information regarding histology, grade, lymph node status, and tumour size was obtained from the medical records and histopathology reports. Vital status was updated as of September 2009 by linking hospital records and the Trent Cancer Registry [13,14].

The Utah Breast Cancer Study (UBCS) identified breast cancer patients from high risk cancer pedigrees, identified through the Utah Population database linked to the Utah Cancer Registry, and excluded cancer patients having *BRCA1* or *BRCA2* mutations. Control subjects comprised distantly related and unrelated cancer-free individuals matched on sex, birth year (within 5 years) and birthplace [15]. The UBCS comprised 898 breast cases and 899 controls.

### TagSNP selection

TagSNPs were selected from the common SNPs [minor allele frequency (MAF) ≧2%] identified from the whole-genome sequencing of 60 unrelated individuals of European ancestry in Utah (CEU low-coverage pilot, 2010-07 release) from the 1000 genomes project [4]. SNPs with Illumina GoldenGate assay design scores of ≧0.8 were given priority as tagSNPs if they were genotyped in the previously reported studies [7–9]. Also, priority was given to SNPs in microRNA target sites as predicted by MicroRNA.org (2010-08 release) [16], since microRNA target SNPs have been shown to affect gene expression by the disruption of the interaction between microRNA and its target mRNA [17]. Aggressive 2-3 marker tagging at r^2^ ≧ 0.8 was used to select tagSNPs based on the hg19 genomic intervals on chromosome 3:142118077-142347668 and chromosome 11: 125445035-125596150 containing the *ATR* and *CHEK1* genes respectively, plus 50kb upstream and downstream of each gene, by use of the Haploview 4.1 software [18]. The initial tagSNP set (32 *ATR* and 40 *CHEK1* tagSNPs) was further supplemented with tags for the known common variants in the exon targeted capture sequencing data on 90 CEU and 43 British from England and Scotland (GBR) from the 1000 genomes project (exon pilot; 2010-03 release) [4]. An additional 8 *ATR* and 10 *CHEK1* tagSNPs were thus identified, resulting in a total of 40 *ATR* and 50 *CHEK1* SNPs for genotyping. The details of the source of tagSNPs and priority (publications or microRNA target sites) are given in Table S1 in File S1.

### Genotyping & quality control

Genotyping was carried out in 96-well microtiter plates using custom Illumina GoldenGate VeraCode assays on the Illumina BeadXpress platform. Genotypes were determined by the genotyping module (1.94) of the Illumina GenomeStudio suite (version 2011.1). Genotyping quality was examined by SNP call rate, duplicate concordance and the Hardy-Weinberg equilibrium (HWE) test in controls. Eight *ATR* and 6 *CHEK1* tagSNPs were excluded from the analysis having call rates of <90%, duplicate concordance of <98%, HWE p value of < 10^-3^ or monomorphism (Table S2 in File S1). Further to this, samples called on < 80% of SNPs were also excluded. The final SBCS data consisted of 32 *ATR* and 44 *CHEK1* tagSNPs on 955 cases and 955 controls. The selected tagSNPs capture 74.9% of ATR and 71.7% of CHK1 SNPs at r^2^ of ≧ 0.9, with mean r^2^ of 88% and 87.4% respectively for SNPs with minor allele frequencies at least 0.02, based on the 1000 genomes 2011-05 release.

### Statistical analysis

#### Imputation

Missing genotypes were imputed for typed SNPs and untyped variants using Impute2 [19,20] based on the 1000 genomes phase I reference panel with singleton variants filtered out. Variants with European MAF ≧ 2% within the *ATR* and *CHEK1* regions were imputed, and included in the subsequent analysis if their imputation information score was greater than 0.8. After filtering, the numbers of eligible imputed variants were 454 and 434 for the *ATR* and *CHEK1* regions, respectively.

#### Breast cancer risk 

Allelic dosage for the minor allele was calculated for typed SNPs and imputed variants, to take account of imputation uncertainty, and was included in a logistic regression model for each SNP. To screen for association with breast cancer risk, allelic odds ratio (OR), 95% confidence interval (CI) and Wald p values were calculated for each SNP using the logistic regression model.

#### Meta analysis 

A literature search was conducted using HuGE Navigator [21]. The terms “*ATR* or *CHEK1*” were used, and the resulting publications were limited to breast neoplasms and gene candidate studies on Europeans. Meta-analysis was then carried out for the published SNPs that were also genotyped in our study. We also included data from a genome-wide association study based on postmenopausal women in Nurses’ Health Study (Cancer Genetic Markers of Susceptibility, CGEMS) [22] if data on relevant SNPs were available. Study specific ORs were pooled by means of both fixed and random models, and the homogeneity of ORs across the centres was evaluated by both Cochran’s Q test and I^2^ measurement implemented in the R metafor package [23].

#### Breast cancer survival 

The Cox proportional hazard model, including age at diagnosis and the left-censoring time between study entry and diagnosis, was employed to estimate the hazard ratio (HR) and 95% CI for each SNP for breast cancer survival. Further adjustment was carried out for lymph node status (binary variable; negative, positive), grade (categorical variable; 1, 2, 3), and tumour size (categorical variable; <2, 2-5, >5 cm) for any significant SNPs. All reported p values are nominal and two-sided. All statistical tests were performed using R 2.15.2.

## Results

### Breast cancer risk 

**Figure 1:**
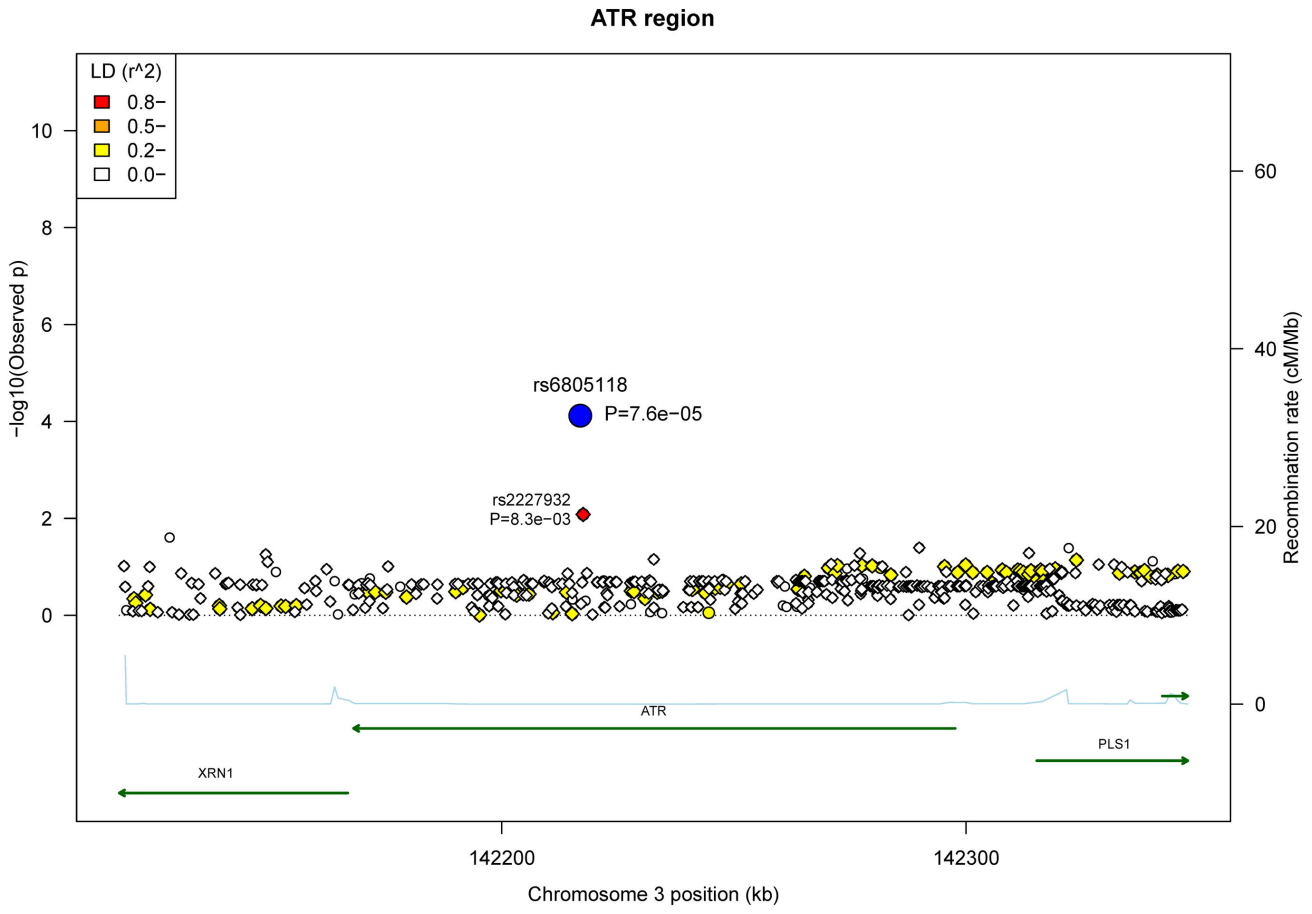
Association plots of the 32 typed SNPs and 454 imputed variants in the *ATR* region. The most significant signal (rs6805118, p=7.6x10^-5^) is labelled with the large blue circle. Circle symbols stand for the typed SNPs and diamond symbols represent the imputed variants. LD associations (r^2^) with rs6805118 were calculated in 85 CEU and 89 GBR (integrated call release as of 2010-11-23) in the 1000 genomes project. Gene transcripts are indicated by the dark green lines, with right arrowhead for the “+” strand and left arrowhead for the “-” strand. Recombination rate (blue line) was obtained from HapMap II.

There are a total of 486 SNPs (32 typed SNPs and 454 imputed variants) in the *ATR* region (229.5 kb) which also contains the *XRN1* and *PLS1* genes. Of these variants, the typed *ATR* SNP rs6805118 yielded the most significant signal for breast cancer risk, with p value of 7.6x10^-5^ (Figure 1). The minor allele of rs6805118 was protective for breast cancer with OR (95% CI) of 0.66 (0.54-0.81) (Table S3 in File S1). The imputed SNP rs2227932, a synonymous *ATR* SNP, in LD with rs6805118 with r^2^ of 0.98 demonstrated a similar protective effect [0.75 (0.61-0.93), p=8.3x10^-3^] (Figure 1 and Table S3 in File S1).

**Figure 2:**
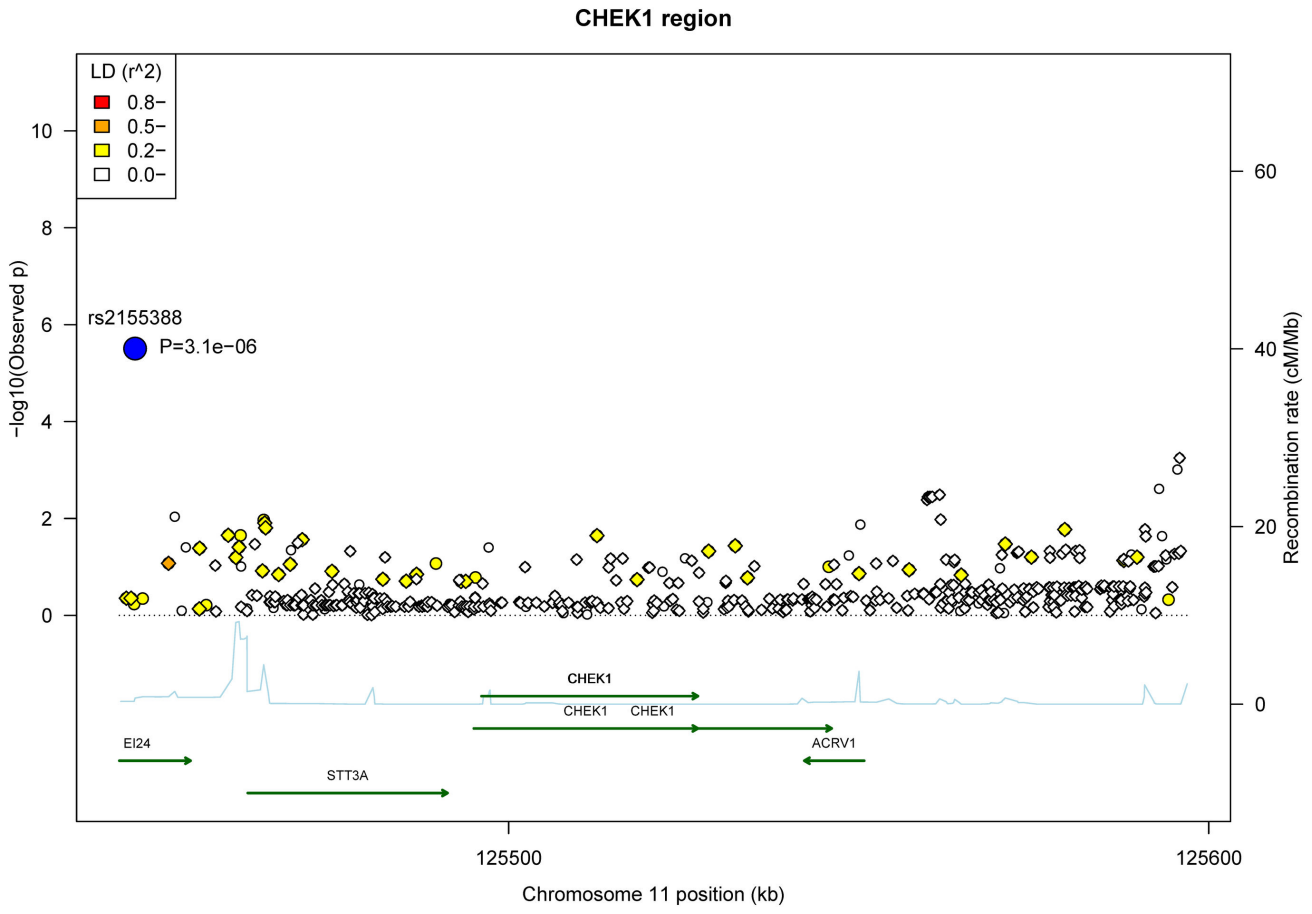
Association plots of the 44 typed SNPs and 434 imputed variants in the *CHEK1* region. The most significant signal (rs2155388, p=3.1x10^-5^) is labelled with the large blue circle. The typed SNPs and the imputed variants are shown in the circle and diamond symbols, respectively. Pair-wise r^2^ with rs2155388 were calculated in 85 CEU and 89 GBR (integrated call release as of 2010-11-23) in the 1000 genomes project. Gene transcripts are indicated by the dark green lines, with right arrowhead for the “+” strand and left arrowhead for the “-” strand. Recombination rate (blue line) was obtained from HapMap II.

For the chromosome 11: 125445035-125596150 region containing the *EI24*, *STT3A*, *CHEK1* and *ACRV1* genes, the most significant association came from the typed rs2155388 SNP at p value of 3.1x10^-6^ (Figure 2), with an allelic effect of OR of 1.43 (95% CI: 1.23-1.65) (Table S4 in File S1). This SNP mapped to the etoposide induced 2.4 (*EI24*) gene. The *EI24* protein is involved in *p53*-mediated apoptosis. There were some other nominally significant associations in the intergenic region telomeric to the *ACRV1* gene (all p ≥5.6x10^-4^).

**Figure 3:**
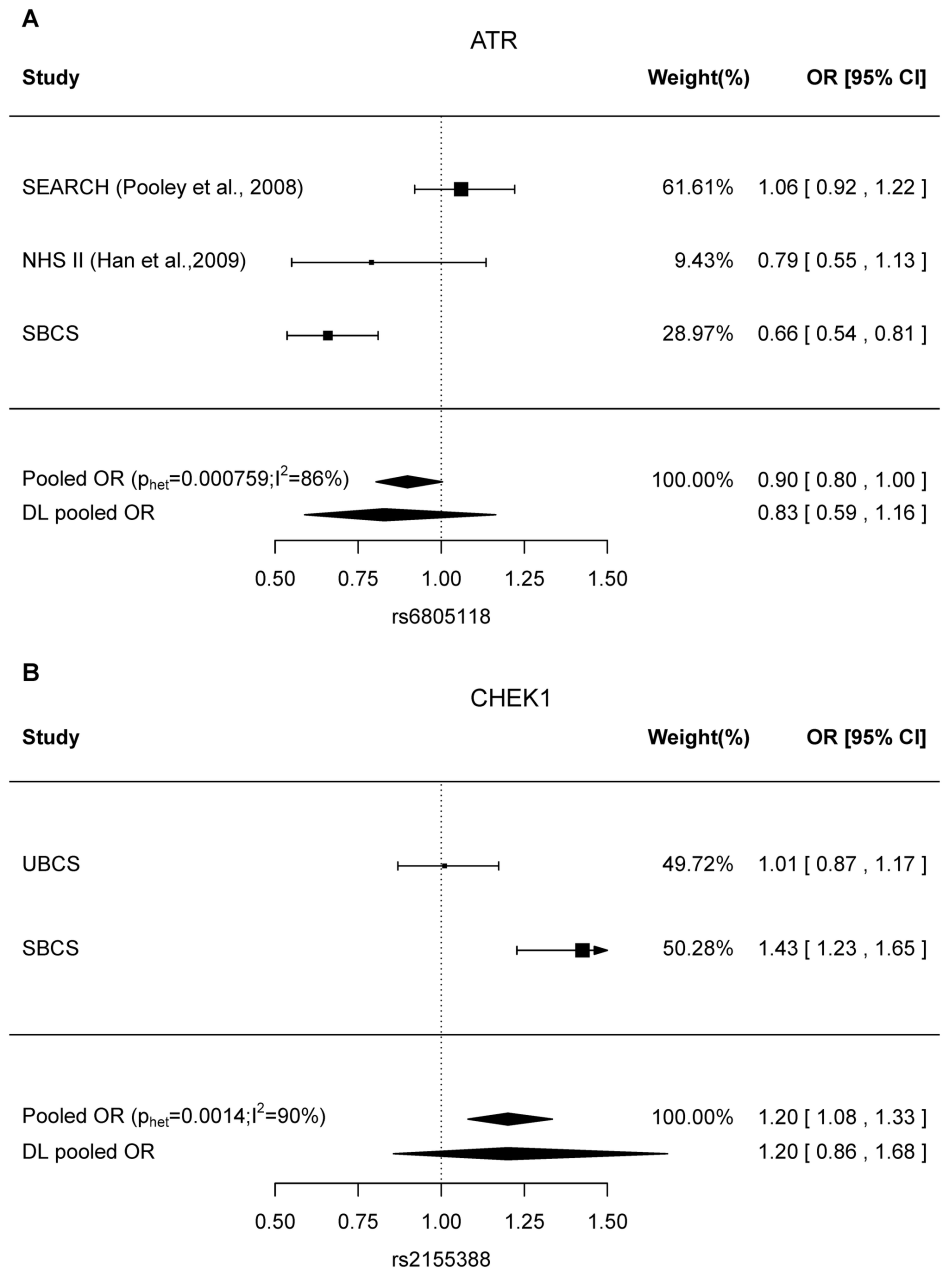
Forest plots of the most significant *ATR* and *CHEK1* SNP associations. (A) the *ATR* rs6805118 associations. SEARCH: Studies of Epidemiology and Risk Factors in Cancer Heredity [8]; NHS II: Nurses’ Health Study (premenopausal women) [9]; SBCS: Sheffield Breast Cancer Study (B) the *CHEK1* rs2155388 associations. UBCS: Utah Breast Cancer Study. For each panel, fixed effect estimate (pooled OR) is shown, with p value for homogeneity (the Cochran’s Q test, p_het_) and I-squared for the amount of heterogeneity in parenthesis. DL pooled OR stands for the random effect model derived from the DerSimonian-Laird estimator.

The top hits in each of the *ATR* and *CHEK1* regions were examined further. The *ATR* SNP rs6805118 was also genotyped in the SEARCH [8] and NHS [9] studies. We therefore conducted a meta-analysis to combine these data and the results are shown in Figure 3A. We found evidence of heterogeneity among the studies (p_het_<1x10^-3^; I^2^=86%). The rs6805118 effect disappeared when all study-specific estimates were combined, with a pooled OR (95% CI) of 0.83 (0.59-1.16) under the random effect model [p=0.277].

The *EI24* SNP rs2155388 in the *CHEK1* region has not been examined in published data. We therefore carried out further genotyping in UBCS, and genotypes were successfully obtained from 859 breast cases and 865 controls. However, the result was not replicated in the UBCS data, with minor allele OR (95% CI) of 1.01 (0.87, 1.17) (Figure 3B). Evidence of heterogeneity between studies was found (p_het_=0.0014, I^2^=90%), and the pooled OR (95% CI) was 1.20 (0.86-1.68) [p=0.29] under the random effect model when both data were combined.

**Figure 4:**
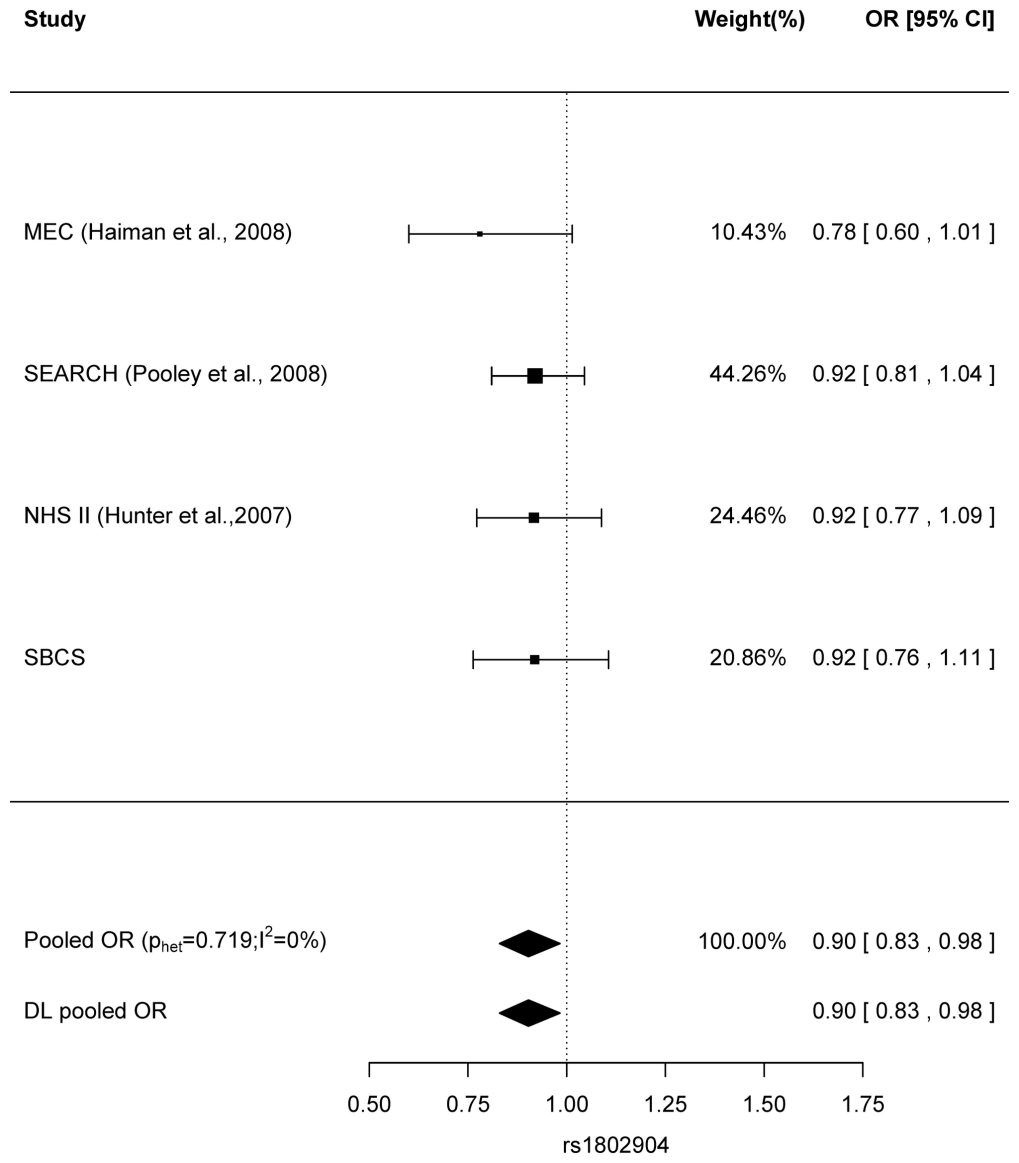
Meta-analysis of the association of rs1802904 (*ATR* region) with breast cancer risk. Fixed effect estimate (pooled OR) is shown, with p value for homogeneity (the Cochran’s Q test, p_het_) and I-squared for the amount of heterogeneity in parenthesis. DL pooled OR stands for the random effect model derived from the DerSimonian-Laird estimator. MEC: Multiethnic Cohort Study [7]; SEARCH: Studies of Epidemiology and Risk Factors in Cancer Heredity [8]; NHS II: Nurses’ Health Study (postmenopausal women) [22]; SBCS: Sheffield Breast Cancer Study.

We also carried out a meta-analysis in an effort to increase power to identify any SNPs with small effects. Published data are available for 11 *ATR* and 11 *CHEK1* SNPs. There was no evidence for effects on breast cancer risk for the majority of these, with all ORs close to unity (Figure S1 for the *ATR* SNPs; Figure S2 for the *CHEK1* SNPs), with the exception of the *ATR* SNP rs1802904 (Figure 4). All studies, including SBCS, showed that the minor allele of rs1802904 was consistently protective for breast cancer by 8-22%, although this association was not statistically significant in any individual study. Study-specific ORs were homogenous for rs1802904 (p_het_=0.719, I^2^=0%), and the pooled OR were the same in both fixed and random effect models, with OR (95% CI) of 0.90 (0.83-0.98) [p=0.0185].

### Breast cancer survival

Vital status post-diagnosis was available for 923 of the 955 SBCS breast cancer subjects. There were 216 deaths during a median of 10.44 years of follow up. Seventy-five percent of the cases are prevalent cases, with a median of 2.32 years between diagnosis and recruitment to study (data not shown). Statistical evidence of survival association was only found for the typed *ATR* SNP rs11920625 (Table S5 in File S1). The HR (95% CI) was 1.37 (1.03-1.81) [p=0.0297] for the minor allele after adjustment for age at diagnosis and accounting for the left-censoring time. This association was no longer significant after further adjusting for lymph node involvement, grade and tumour size (p=0.064, Table S5 in File S1), and showed a pooled HR (95% CI) of 1.18 (0.91-1.55) [p=0.21; data not shown] when combining with a HR of 1.04 from the SEARCH study [8]. There was no evidence of association between SNPs in the *CHEK1* region and breast cancer overall survival (Table S6 in File S1).

## Discussion

### *ATR* in risk and survival 

We identified an association between *ATR* rs6805118 and breast cancer risk in the SBCS discovery set. A protective effect of the minor allele of rs6805118 was also found in the NHS premenopausal women [9], however the minor allele conferred an increased risk of breast cancer in SEARCH subjects [8]. Meta-analysis of these 3 datasets revealed a high degree of heterogeneity and non-significant pooled OR, suggesting little overall evidence for association with breast cancer risk. In addition, neither SEARCH nor the current study found any evidence for the association of this SNP with survival [8].

The *ATR* synonymous rs1802904 SNP was found to be nominally significantly associated with breast cancer risk (p=0.0185) by means of the meta-analysis of the MEC European Americans [7], NHS postmenopausal women [22], SEARCH [8] and SBCS data. There was no evidence for heterogeneity (I^2^=0). This SNP lies in the target region of hsa-miR-27a and hsa-miR-27b [16], and in regions of histone modification marks (H3k4me3 and H3k27ac). Therefore, this SNP might exert its effect on breast cancer susceptibility via transcriptional or post-transcriptional gene regulation. Although we cannot rule out the possibility that this is a false positive, further replication is warranted in a larger study.

### *CHEK1* region in risk and survival 

The chromosome 11 interval containing the *EI24* and *CHEK1* genes is a frequently altered region in breast cancer. A molecular study of breast cancer tumour samples revealed a high frequency of deletions and promoter methylations in the *EI24* and *CHEK1* genes, and reported poor survival associations for patients with these alterations [24]. These findings highlight the important roles of acquired deletions and inherited epigenetic events of the *EI24* and *CHEK1* genes in breast cancer progression. The SBCS finding of an rs2155388 effect was not replicated in the UBCS, despite that fact that UBCS had an adequate power of 90% to detect similar associations. Null results were also obtained in our survival analysis. As a result, there was little evidence in our study supporting any inherited sequence variants in the *CHEK1* region being associated with breast cancer risk or survival.

In this study, while we can rule out moderate effects of the less common/rare alleles, we are unable to exclude weaker effects of these, for example, odds ratios of less than 1.46 for SNPs with MAF of 5% or less, at 80% power. However, we conclude there is little evidence to support any role for common SNPs in the *ATR* and *CHEK1* regions in breast cancer risk or survival.

## Supporting Information

Figure S1 Meta-analysis of the typed SNPs in the *ATR* region. For each panel, fixed effect estimate (pooled OR) is shown, with p value for homogeneity (the Cochran’s Q test, p_het_) and I-squared for the amount of heterogeneity in parenthesis. DL pooled OR stands for the random effect model derived from the DerSimonian-Laird estimator. MEC: Multiethnic Cohort Study [7]; SEARCH: Studies of Epidemiology and Risk Factors in Cancer Heredity [8]; NHS II: Nurses’ Health Study (Han et al., 2009; premenopausal women) [9]; NHS II: Nurses’ Health Study (Hunter et al., 2007; postmenopausal women) [22]; Spain (Barroso et al., 2009) [12]; Cyprus (Loizidou et al., 2010) [11]; SBCS: Sheffield Breast Cancer Study.
(TIFF)

Figure S2 Meta-analysis of the typed SNPs in the *CHEK1* region. For each panel, fixed effect estimate (pooled OR) is shown, with p value for homogeneity (the Cochran’s Q test, p_het_) and I-squared for the amount of heterogeneity in parenthesis. DL pooled OR stands for the random effect model derived from the DerSimonian-Laird estimator. MEC: Multiethnic Cohort Study [7]; SEARCH: Studies of Epidemiology and Risk Factors in Cancer Heredity [8]; NHS II: Nurses’ Health Study (Han et al., 2009; premenopausal women) [9]; NHS II: Nurses’ Health Study (Hunter et al., 2007; postmenopausal women) [22]; Cyprus (Loizidou et al., 2010) [11]; SBCS: Sheffield Breast Cancer Study.
(TIFF)

File S1 File **S1**.
(XLS)
